# Genetic Insights into Hemiplegic Migraine: Whole Exome Sequencing Highlights Vascular Pathway Involvement via Association Analysis

**DOI:** 10.3390/genes16080895

**Published:** 2025-07-28

**Authors:** Zizi Molaee, Robert A. Smith, Neven Maksemous, Lyn R. Griffiths

**Affiliations:** 1 Genomics Research Centre, School of Biomedical Sciences, Faculty of Health, Queensland University of Technology, 60 Musk Ave, Kelvin Grove, QLD 4059, Australia; zizi.molaee@hdr.qut.edu.au (Z.M.); r157.smith@qut.edu.au (R.A.S.); n.maksemous@qut.edu.au (N.M.); 2 Genomics Research Centre, Research Infrastructure, Faculty of Sciences, Queensland University of Technology, 60 Musk Ave, Kelvin Grove, QLD 4059, Australia

**Keywords:** hemiplegic migraine, whole exome sequencing, small vessel disease, genetic variants, *LRP1*, *COL4A1*, *COL4A2*, *TGFBR2*, migraine genetics, vascular dysfunction

## Abstract

**Background**: Hemiplegic migraine (HM) is a rare and severe subtype of migraine with a complex genetic basis. Although pathogenic variants in *CACNA1A*, *ATP1A2*, and *SCN1A* explain some familial cases, a significant proportion of patients remain genetically undiagnosed. Increasing evidence points to an overlap between migraine and cerebral small vessel disease (SVD), implicating vascular dysfunction in HM pathophysiology. **Objective**: This study aimed to identify rare or novel variants in genes associated with SVD in a cohort of patients clinically diagnosed with HM who tested negative for known familial hemiplegic migraine (FHM) pathogenic variants. **Methods**: We conducted a case-control association analysis of whole exome sequencing (WES) data from 184 unrelated HM patients. A targeted panel of 34 SVD-related genes was assessed. Variants were prioritised based on rarity (MAF ≤ 0.05), location (exonic/splice site), and predicted pathogenicity using in silico tools. Statistical comparisons to gnomAD’s Non-Finnish European population were made using chi-square tests. **Results**: Significant variants were identified in several SVD-related genes, including *LRP1* (p.Thr4077Arg), *COL4A1* (p.Pro54Leu), *COL4A2* (p.Glu1123Gly), and *TGFBR2* (p.Met148Leu and p.Ala51Pro). The *LRP1* variant showed the strongest association (*p* < 0.001). All key variants demonstrated pathogenicity predictions in multiple computational models, implicating them in vascular dysfunction relevant to migraine mechanisms. **Conclusions**: This study provides new insights into the genetic architecture of hemiplegic migraine, identifying rare and potentially deleterious variants in SVD-related genes. These findings support the hypothesis that vascular and cellular maintenance pathways contribute to migraine susceptibility and may offer new targets for diagnosis and therapy.

## 1. Introduction

Migraine is a highly prevalent neurological disorder, affecting more than one billion people worldwide. It is ranked as the second leading cause of years lived with disability (YLDs) globally, and the primary cause of disability among women under 50 years of age [1]. The condition disproportionately affects females, with a female-to-male ratio of approximately 3:1, highlighting possible hormonal and genetic contributions [2]. Twin studies estimate that genetic factors account for up to 60% of migraine heritability, although its underlying biology remains complex and incompletely understood [3]. Migraine also exhibits a range of inheritance patterns, ranging from rare monogenic forms like Familial Hemiplegic Migraine (FHM), with a prevalence of approximately 0.01% [4], to more common polygenic types [5].

Familial Hemiplegic Migraine (FHM) is a rare autosomal dominant subtype of migraine with aura, characterised by transient motor weakness (hemiparesis) during attacks, and often accompanied by visual, sensory, or language disturbances. It is clinically defined by the presence of at least one first- or second-degree relative with similar symptoms [6]. FHM is known to be caused by pathogenic variants in *CACNA1A*, *ATP1A2*, and *SCN1A* [7], but these variants only explain about half of FHM cases, suggesting that additional genetic factors are involved. There have been indications of links between migraine and small vessel disease (SVD) of the brain [8,9,10]. Studies have shown that individuals with migraine, particularly migraine with aura, have a higher prevalence of silent brain infarcts and white matter hyperintensities, which are markers of SVD [8,11]. Genome-wide association studies (GWAS) have further reinforced this relationship, identifying susceptibility loci involving both neuronal and vascular mechanisms, including genes related to endothelial integrity and blood–brain barrier regulation [4,12,13].

While classical FHM has been associated with neuronal ion channel dysfunction, recent findings suggest that vascular mechanisms may also play a role in migraine pathophysiology [14]. Disruption of the blood–brain barrier [15], endothelial dysfunction [16], and altered cerebral autoregulation [9] have all been implicated. Therefore, it is plausible that rare variants in SVD-related genes contribute to migraine with aura susceptibility, particularly in cases where no known pathogenic variants in ion channels are identified.

This study aimed to identify potentially causal variants in SVD-related genes in HM individuals without known causative variants using whole exome sequencing (WES) [17]. We hypothesised that such variants would be present due to the potential difficulties in making an accurate diagnosis by symptoms alone, where SVD without a significant stroke event may appear as a phenocopy of FHM, as post-stroke symptoms mimic the hemiplegia associated with hemiplegic migraine.

Our research aimed to expand the list of genes implicated in hemiplegic migraine and contribute to a broader understanding of its pathophysiology. By identifying new genetic factors, we aimed to improve diagnostic strategies and potentially reveal novel therapeutic targets for migraine treatment.

## 2. Methods

### 2.1. Study Population

This study was conducted at the Genomics Research Centre (GRC), Queensland University of Technology (QUT), Brisbane, Australia. It included 184 unrelated individuals clinically diagnosed, by consultant neurologists, with hemiplegic migraine based on the International Classification of Headache Disorders (ICHD) criteria [6]. Notably, this HM cohort represents one of the largest genetically screened hemiplegic migraine cohorts published to date. Participants were referred to the Genomics Research Centre (GRC) for molecular diagnostics testing. All subjects had previously tested negative for pathogenic variants in the established FHM genes (*CACNA1A*; *ATP1A2*; and *SCN1A*) [17,18,19,20], as well as other FHM-related genes (including *PRRT2*; *PNKD*; *SLC1A3*; *SLC2A1*; *SLC4A4*; *ATP1A3*; and *ATP1A4*) [17,18,19,20,21].

This study was conducted as a case-control genetic association analysis, aimed at identifying novel contributors to hemiplegic migraine. All participants were referred by neurologists and headache clinics across Australia and New Zealand. Family history data and clinical information beyond the HM diagnosis remain limited for this cohort [21]. Ethnic background was assessed through clinical records, and all individuals included in the final analysis were of confirmed Caucasian (non-Finnish European) descent, to match the reference population used in gnomAD comparisons. Although migraine prevalence differs by sex, a sex-specific analysis was not conducted in this study.

Inclusion criteria were as follows: (1) a clinical diagnosis of hemiplegic migraine made by a consultant neurologist; (2) an absence of pathogenic variants in known FHM genes or FHM-related genes; and (3) availability of sufficient quality genomic DNA for sequencing. Exclusion criteria included the following: (1) known secondary causes of hemiplegia (e.g., stroke, tumour); (2) detection of pathogenic variants in known FHM genes or FHM-related genes, either through prior testing or during the study; and (3) low DNA yield or quality incompatible with whole exome sequencing.

### 2.2. Whole Exome Sequencing

Genomic DNA, from the 184 index cases previously referred to the GRC for molecular genetics testing, was extracted from peripheral blood samples either in-house using the QIAGEN QIAcube (Clayton, VIC, Australia) or was extracted and purified by referring hospitals. Whole exome sequencing was performed using the Ion Torrent platform with the Ion AmpliSeq Exome RDY kit (Thermo Fisher Scientific, Carlsbad, CA, USA) (covering around 20,000 genes). NGS libraries for WES were constructed following the manufacturer’s protocol. The Ion Chef system (Thermo Fisher Scientific, Carlsbad, CA, USA) was used for template preparation and loading of the barcoded sample library fragments onto the chips. Sequencing was performed on Ion Proton and GeneStudio S5 Plus instruments (Thermo Fisher ScientificThermo, Carlsbad, CA, USA) at the Genomics Research Centre (GRC) in Australia, using default settings for the Ion AmpliSeq Exome RDY Kit 4 × 2. Data from sequencing were processed and aligned using the Ion Torrent server v5.10 (Thermo Fisher Scientific, Carlsbad, CA, USA). Variant annotation and data analysis were performed using Ion Reporter software (Thermo Fisher Scientific, Carlsbad, CA, USA) v5.12.

### 2.3. Data Analysis Pipeline

#### 2.3.1. Targeted Gene Selection

A comprehensive panel of 34 genes associated with SVD, vascular integrity, and cerebrovascular pathology was selected based on an extensive review of the literature [22], online databases, e.g., NCBI (https://www.ncbi.nlm.nih.gov/omim) accessed on 3 October 2022, and prior Genome-wide association (GWAS) and sequencing studies. Genes were selected if they met one or more of the following criteria: (1) reported pathogenic variants known to cause SVD; (2) involvement in vascular function, endothelial regulation, or cellular maintenance based on pathway analyses; and (3) association with stroke-like phenotypes in the published literature. This structured approach allowed us to prioritise genes with a high biological plausibility for vascular involvement in HM. The primary FHM genes were also included to verify that no known FHM causative variants were present. This gene panel included *NOTCH3*, *APP*, *FOXC1*, *LRP1*, *SLC2A1*, *HTRA1*, *CDKN2B-AS1*, *CACNA1A*, *SLC1A3*, *MT-TL1*, *ATP1A2*, *WASL*, *SLC4A4*, *LMOD2*, *MTDH*, *CST3*, *CBS*, *PHACTR1*, *COLGALT1*, *SCN1A*, *CTC1*, *CTSA*, *CDKN2B*, *PRRT2*, *ACE*, *CDKN2A*, *TREX1*, *MTHFR*, *KCNK18*, *TGFBR2*, *PNKD*, *COL4A2*, *COL4A1*, and *GLA*. These genes are involved in various molecular pathways for vascular function, cellular maintenance, and metabolism, reflecting the complex aetiology of SVD and related cerebrovascular disorders.

#### 2.3.2. Initial Filtering

In the initial filtering stage, a custom filter was applied to the whole exome sequencing data of the 184 hemiplegic migraine subjects. This filter was designed to focus on rare variants in 34 pre-selected genes associated with SVD and cerebrovascular conditions. Variants were also excluded on the basis of allele frequency, with only variants with a minor allele frequency (MAF) ≤ 0.05 considered for further analysis. The filter is additionally selected for variants in exonic regions or splice sites, including missense variants, nonsense variants, and small insertions or deletions that could potentially impact protein function. Our focus on rare variants was based on the hypothesis that unsolved cases of hemiplegic migraine are more likely to be driven by low-frequency and high-impact genetic changes. While common variants may contribute to polygenic risk, the aim of this study was to identify novel rare contributors in a genetically unexplained monogenic-like cohort.

#### 2.3.3. Data Merging and Exclusion

After the initial filtering, the variant data from all 184 participants were combined into a single dataset. Variants involving large deletions and repeats expansion were excluded due to the inability of our method to reliably detect them. Instead, we focused on the small genetic changes (SNVs and small indels) that our technology can reliably identify. However, we acknowledge that larger genetic variations may also play a significant role and could be explored in future research.

#### 2.3.4. Quality Control and Classification

Following annotation, the filtered variants were visually inspected using the Integrative Genomics Viewer (IGV) [23] to confirm variant calls and eliminate systemic errors that had escaped automated quality control assessment. Variants with quality scores of below 30, read depths of less than 20, and inconsistent read support (e.g., reads only on one strand) were also excluded. Functional impacts were predicted using the in silico tools of REVEL [24], CADD [25], SIFT [26], Mutation Taster [27], and PolyPhen-2 [28] scores. Variants were cross-referenced against the ClinVar [29] and dbSNP [30] databases, as well as in the literature, to assess previous disease associations.

Variants predicted to be damaging by multiple algorithms were prioritised; variants with inconclusive or mixed predictions were not dismissed. The pathogenicity of each variant was evaluated using five established in-silico models: REVEL, CADD, SIFT, Mutation Taster, and PolyPhen-2. No single model was weighted above others; instead, variants were considered “likely damaging” when they were classified as deleterious or disease-causing by at least three out of five models, and “inconclusive” when predictions were mixed or borderline. REVEL and CADD scores above established thresholds (REVEL > 0.5; CADD > 20) were used as strong indicators of functional impact. These predictions were further cross-referenced with ClinVar and literature evidence. A gene was prioritised for interpretation if at least one variant met these criteria and showed statistically significant over-representation in the HM cohort compared to the control dataset. We acknowledge that in silico tools have limitations, particularly in detecting tissue-specific or regulatory effects, and that some variants may exert their influence in ways not captured by current models. As such, all variants meeting the quality and frequency thresholds were retained for further analysis, with a view to guiding future functional investigations and clinical interpretation.

### 2.4. Statistical Analysis

To evaluate the significance of the identified variants, a statistical analysis was conducted comparing the frequency of each variant in the hemiplegic migraine (HM) cohort to the frequency in a control population, using chi-square (χ^2^) tests. Control population data were obtained from the Non-Finnish European (NFE) subset of the gnomAD database, which was selected to closely match the confirmed Caucasian ancestry of our study cohort. The NFE dataset provided the most appropriate and statistically robust comparator available for allele frequency analysis in this context. The frequency of each variant in the HM cohort was organised into tables and compared to the variant frequency in the control population using chi-square tests to determine whether specific variants were significantly over-represented in the HM cohort compared to the control group. The minor allele frequency (MAF) for each variant was calculated as the proportion of minor alleles observed in the total number of alleles screened across the cohort (i.e., 2n for autosomal loci). This value was compared to the MAF from the reference population (gnomAD NFE), as listed next to the corresponding nucleotide change in Table 1. Variants with expected cell counts < 5 were reported but interpreted cautiously due to potential statistical instability. The significance threshold for these tests was set as α = 0.05.

## 3. Results

Our analysis identified several significant genetic variants in SVD-related genes, which were examined for their potential role in hemiplegic migraine. These variants are summarised in Table 1, which includes minor allele frequencies (MAF), dbSNP identifiers, and amino acid changes. One of the most notable findings was the *LRP1* p.Thr4077Arg (c.12230C>G) variant, which was observed in 3 out of 184 hemiplegic migraine patients (MAF = 0.008), compared to MAF = 0.00077 in the Non-Finnish European (NFE) population. Chi-square analysis, presented in Table 2, showed a highly significant association for this variant with a chi-square value of 25.11 and a *p*-value of 5.41 × 10^−7^. Additionally, the *COL4A1* p.Pro54Leu (c.161C>T) variant was found in four patients (MAF = 0.01) and showed a significant association (χ^2^ = 3.88, *p* = 0.049). In silico prediction tools further support their pathogenic potential, suggesting deleterious effects on gene function (Table 1). These findings suggest that these variants may impair vascular integrity, contributing to the development of hemiplegic migraine.

In addition to *LRP1* and *COL4A1/2*, we identified multiple rare variants in *TGFBR2*, a gene involved in vascular regulation. Of the *TGFBR2* variants, p.Met148Leu was exclusive to the hemiplegic migraine cohort. The *TGFBR2* variant, p.Ala51Pro, showed a high level of significance in the statistical analysis (Table 2). Although these variants are rare, occurring in single patients, the role of *TGFBR2* in the TGF-beta signalling pathway, which regulates vascular integrity and inflammation, suggests they may contribute to vascular instability in these migraine patients.

## 4. Discussion

Our study identified rare variants in genes involved in SVD and vascular regulation in our hemiplegic migraine cohort (n = 184), many of which may contribute to hemiplegic migraine (HM). Among these, the *CTC1* p.Gly414Ala variant (found in 5 patients) and the *CDKN2A* p.Ala148Thr variant (identified in 13 patients) were also identified in a separate whole exome trio analysis of migraine families (unpublished data), suggesting a shared genetic basis for migraine susceptibility across different forms of migraine. Despite this, the *CTC1* variant showed no significant association (χ^2^ = 0.50, *p* = 0.478), and the *CDKN2A* variant also failed to show statistical significance (χ^2^ = 0.06, *p* = 0.800). These genes, involved in telomere maintenance and cell cycle regulation [31,32,33], may influence vascular or neuronal pathways implicated in migraine.

The *LRP1* p.Thr4077Arg variant (c.12230C>G) demonstrated strong statistical association in our study (χ^2^ = 25.11, *p* = 5.41 × 10^−7^), being found in three patients (MAF = 0.008) compared to its extremely low frequency in the Non-Finnish European population (MAF = 0.00077). *LRP1* (Low-density lipoprotein receptor-related protein 1) is a multifunctional cell surface receptor involved in various biological processes, including lipid metabolism, cell signalling [34], and blood-brain barrier integrity [35]. The p.Thr4077Arg variant is located in a highly conserved region of the protein and shows strong evidence of pathogenicity with a CADD score of 24.2. Given *LRP1*’s role in maintaining blood-brain barrier integrity and its involvement in neuroinflammatory responses, this variant could potentially disrupt these processes, contributing to migraine pathophysiology. The significant over-representation of this variant in our hemiplegic migraine cohort suggests it may be an important risk factor for the condition.

Associations were also observed in collagen IV genes. The *COL4A1* variant (c.161C>T, p.Pro54Leu) was identified in four patients (MAF = 0.01) compared to a MAF = 0.004205 in the NFE population, with a modest but statistically significant chi-square result (χ^2^ = 3.88, *p* = 0.049). In contrast, the *COL4A2* p.Glu1123Gly variant, though more common in the HM cohort (n = 8, MAF = 0.022), did not reach statistical significance (χ^2^ = 2.41, *p* = 0.121). In silico predictors suggest a damaging effect for both variants (see Table 1), but the statistical support is stronger for *COL4A1*. These findings suggest that the *COL4A1* variant contributes to functional impairment in collagen proteins, potentially linking hemiplegic migraine attacks to vascular dysfunction. COL4A1 is a type IV collagen protein, and variants in it are known to cause significant vascular disease, with the mechanism believed to be disruption of the usual conformation of the protein heterotrimer it forms with COL4A2, resulting in weakened collagen and vascular integrity [36,37,38]. Although the p.Pro54Leu variant is not the typical pathogenic *COL4A1* variant, consisting of a loss of a glycine in an alpha helix domain [37,39], the variant may still affect protein folding and interactions sufficiently to cause some degree of weakened vascular integrity. This vascular instability may also increase neuronal excitability by releasing signalling molecules from damaged cells, contributing to migraine symptoms.

The *COL4A1* p.Pro54Leu variant is located in the 7S domain of COL4A1, which is essential for the assembly of the collagen IV network. The variant was previously reported to be heterozygous in an individual with unilateral optic nerve hypoplasia and was inherited from a father with a history of eye problems [40]. Moreover, p.Pro54Leu was identified in another study as a common, highly functional missense variant in both US and Scottish datasets, but the study did not provide a detailed analysis of its specific impact on intracerebral haemorrhage risk [41]. However, to our knowledge, no functional studies have been conducted specifically on this variant. Further protein structural or functional studies are needed to investigate this variant’s impact on COL4A1 function and migraine mechanisms. The link between *COL4A1* variants and migraine may arise from vascular dysfunction, blood-brain barrier (BBB) disruption, and neuronal excitability. Pathogenic variants in exon 3 could weaken vascular integrity, leading to abnormal cerebral blood flow, a known trigger for migraine [42]. Additionally, *COL4A1* pathogenic variants are associated with SVD, which shares features with migraine [43,44].

The *TGFBR2* variants in our study showed some of the strongest statistical associations among all identified variants. Two rare variants were identified in *TGFBR2*, including p.Ala51Pro and a novel variant, p.Met148Leu. The p.Ala51Pro variant showed a highly significant association with hemiplegic migraine (χ^2^ = 99.45, *p* = 2.01 × 10^−23^). However, the very low allele counts limit the effectiveness of this analysis. The p.Met148Leu variant, while observed in the cohort, was completely absent in the NFE population, and therefore could not be included in the final chi-square analysis. The p.Ala51Pro variant showed an extremely low frequency in the NFE population (MAF = 0.00001798). *TGFBR2* encodes a receptor in the TGF-beta signalling pathway, which plays a crucial role in maintaining vascular integrity and inflammation [45]. Given the established association between migraine and vascular instability and inflammation [46], the pathogenic variants in *TGFBR2* may contribute to vascular dysfunction [47,48], potentially increasing susceptibility to migraine. A previous study investigating *TGF-β1* polymorphisms in pediatric migraine reported differences in variant distribution, further supporting the involvement of vascular and inflammatory mechanisms [49]. Despite their rarity, these variants could still influence migraine pathophysiology through vascular pathways.

Our study, utilising the largest known hemiplegic migraine cohort to date, shows an overlap between hemiplegic migraine and SVD genes, highlighting a potential shared genetic basis. This expands our understanding of migraine’s genetic landscape and its links to cerebrovascular disorders, offering potential targets for drug development and risk assessment. The pathophysiology of migraine aura, including hemiplegic migraine, is thought to involve cortical spreading depression (CSD), disruption of ion homeostasis, and activation of the trigeminovascular system. These mechanisms result in sterile neuroinflammation, vasodilation, and transient neuronal dysfunction. The variants identified in this study, such as in *COL4A1*, *TGFBR2*, and *LRP1,* are known to influence vascular stability, endothelial signalling, and blood–brain barrier permeability. Disruption of these systems may amplify neuronal excitability or impair neurovascular coupling, thereby facilitating CSD and increasing susceptibility to hemiplegic migraine attacks. These findings reinforce a neurovascular model of HM pathogenesis, integrating both vascular and neuronal dysfunction.

Future research should focus on prioritising functional studies of identified variants in relevant cell lines, organoids, or animal models to assess their potential mechanisms of action. Additionally, replication of these findings in diverse populations is also essential to evaluate the influence of ethnicity and lifestyle factors. Another direction for future research is to compare the prevalence and significance of SVD-associated variants in hemiplegic migraine patients with those in unselected or polygenic migraine populations, which may help to refine the specificity of these genetic associations. In the longer term, exploration of non-coding regions may provide further insight into the role of SVD genes in migraine pathophysiology. A limitation of this study is the lack of uniformly available clinical data, including imaging, EEG, lumbar puncture results, and formal exclusion of alternative diagnoses, across the entire cohort.

## 5. Conclusions

This study presents novel evidence linking hemiplegic migraine (HM) to rare variants in genes previously implicated in cerebral SVD, including *LRP1*, *COL4A1*, and *TGFBR2*. By identifying potentially deleterious variants in a large, clinically diagnosed HM cohort, this study highlights a possible alternative mechanism contributing to HM pathophysiology, extending beyond the classical focus on ion channel dysfunction. These findings not only expand the known genetic spectrum of HM but also support the development of broader, clinically relevant diagnostic gene panels. Future research should aim to validate the functional impact of these variants and explore their relevance in other migraine subtypes.

## Figures and Tables

**Table 1:** Summary of genetic variants identified in hemiplegic migraine patients and their frequency in gnomAD NFE.

Gene	Locus	dbSNP	Amino Acid Change	Coding	Transcript	Revel	CADD	SIFT	Polyphen-2	Mutation Taster
*LRP1*	chr12:57602950	rs142667784	p.Thr4077Arg	c.12230C>G	NM_002332.2	0.632	24.2	0.011	0.998	D
*COL4A1*	chr13:110866346	rs34004222	p.Pro54Leu	c.161C>T	NM_001845.5	0.746	29.9	0.03	0.999	D
*COL4A2*	chr13:111143601	rs117412802	p.Glu1123Gly	c.3368A>G	NM_001846.3	0.468	25.2	0.04	0.707	D
*CTC1*	chr17:8138569	rs62624978	p.Gly414Ala	c.1241G>C	NM_025099.5	0.264	22	0.192	ND	D
*NOTCH3*	chr19:15290236	rs112197217	p.His1133Gln	c.3399C>A	NM_000435.2	0.438	6.878	0.002	ND	P
*CDKN2A*	chr9:21970916	rs3731249	p.Ala148Thr	c.442G>A	NM_001195132	0.271	14.75	0.011	0.804	P
*MTHFR*	chr1:11850750	rs35737219	p.Thr653Met	c.1958C>T	NM_005957.4	0.139	1.795	0.38	0.002	P
*LRP1*	chr12:57539082	rs1800127	p.Ala217Val	c.650C>T	NM_002332.2	0.263	24.3	0.02	0.003	P
*TGFBR2*	chr3:30664747	rs557449314	p.Ala51Pro	c.151G>C	NM_001024847.2	0.194	11.66	0.3	ND	P
*TGFBR2*	chr3:30691865	rs768385200	p.Met148Leu	c.442A>T	NM_001024847.2	0.645	23.5	0	0.314	D
*TGFBR2*	chr3:30713834	rs35766612	p.Val412Met	c.1234G>A	NM_001024847.2	0.79	25	0.115	0.999	P
*GLA*	chrX:100653420	rs28935490	p.Asp313Tyr	c.937G>T	NM_000169.2	0.614	18.33	0.001	0.999	P
*CBS*	chr21:44480591	rs117687681	p.Arg369Cys	c.1105C>T	NM_001178008.2	0.972	31	0	ND	D

REVEL: Scores closer to 1 suggest a higher likelihood of pathogenicity, CADD: Scores above 20 suggest a higher likelihood of pathogenicity, SIFT: Scores below 0.05 indicate a higher likelihood of damaging effects, PolyPhen-2: Scores closer to 1 indicate a higher likelihood of the variant being damaging, Mutation Taster: D: Disease-causing, P: Polymorphism, ND: No data.

**Table 2:** Chi-Square Analysis of Variants Identified in Hemiplegic Migraine (HM) Compared to Non-Finnish European (NFE) Population.

Gene	Variant and Locus	Chi-Square	*p*-Value	df	MAF in the HM Cohort	gnomAD NFE
*LRP1*	chr12:57602950-C>G *	25.11	5.41 × 10^−7^	1	0.008	0.0007750
*COL4A1*	chr13:110866346-G>A *	3.88	0.049	1	0.01	0.004205
*COL4A2*	chr13:111143601-A>G	2.41	0.121	1	0.022	0.01267
*CTC1*	chr17:8138569-C>G	0.50	0.478	1	0.014	0.01859
*NOTCH3*	chr19:15290236-G>T	0.02	0.877	1	0.019	0.01795
*CDKN2A*	chr9:21970916-C>T	0.06	0.800	1	0.035	0.03296
*MTHFR*	chr1:11850750-G>A	0.39	0.530	1	0.016	0.02101
*LRP1*	chr12:57539082-C>T	0.00	0.957	1	0.0249	0.02490
*TGFBR2*	chr3:30664747-G>C *	99.45	2.01 × 10^−23^	1	0.003	0.00001798
*TGFBR2*	chr3:30691865-A>T	N/A	N/A	-	0.003	ND
*TGFBR2*	chr3:30713834-G>A	0.11	0.740	1	0.003	0.001954
*GLA*	chrX:100653420-C>A	0.25	0.617	1	0.003	0.004456

Abbreviations: df: degrees of freedom, NFE: Non-Finnish European gnomAD population. *: Variant significance lower than a = 0.05 threshold, ND: no data.

## Data Availability

The sequencing data presented in this study are available on request from the corresponding author.
